# Hesperidin alleviated D-GalN/LPS induced acute liver injury in mice: insights into gut microbiota and the Nrf2/Keap1 pathway

**DOI:** 10.3389/fmicb.2025.1725863

**Published:** 2025-12-17

**Authors:** Kaiming Li, Gaohuan Hou, Hongbin Deng, Weiting Lu, Hongyou Liu, Weiqin Li, Qi Wang, Zhihui Tong

**Affiliations:** 1 Jinling Clinical Medical College, Nanjing University of Chinese Medicine, Nanjing, China; 2 School of Medicine, Southeast University, Nanjing, Jiangsu, China; 3 Department of Critical Care Medicine, Jinling Hospital, Affiliated Hospital of Medical School, Nanjing University, Nanjing, China

**Keywords:** hesperidin, acute liver injury, gut microbiota, gut-liver axis, Nrf2/Keap1 signaling pathway

## Abstract

**Background:**

Acute liver injury (ALI) has grown to be a major worldwide health issue, yet current therapeutic approaches remain inadequate. A growing number of studies suggest that hesperidin demonstrates high therapeutic potential in the treatment of acute liver injury. However, the specific mechanisms by which hesperidin alleviates ALI remain unclear. Herein, we aimed to investigate how hesperidin ameliorates acute liver injury under conditions that influence the gut microbiota.

**Methods:**

A mouse model of ALI was established by D-GalN/LPS injection, and 16S rDNA sequencing was employed to explore the underlying mechanism of hesperidin in alleviating ALI. Forty mice were divided into the control group (CON), the LPS&D-GalN group (ALI), the HDN group (feed hesperidin), the HDA group (the positive drug group, feed hesperidin). Liver, ileum, and serum samples were collected randomly from five mice in each of the four groups to evaluate liver injury markers, inflammatory indicators, intestinal barrier function indices, and antioxidant parameters. Additionally, cecum contents were collected for 16S rDNA sequencing to analyze the gut microbiota.

**Results:**

Hesperidin significantly ameliorated D-GalN/LPS-induced abnormal transaminase activities, liver and intestinal systemic inflammation, and intestinal environmental disorders. Furthermore, activation of the Nrf2/Keap1 signal pathway by HDN reduced the production of proinflammatory cytokines (TNF-α, IL-1β and IL-6) and boost the activity of antioxidant enzymes (CAT and SOD). HDN restored SCFAs to normal levels by upregulating the abundance of beneficial bacteria (Lachnospiraceae_NK4A136_group, *Alloprevotella* and *Clostridia_UCG-014*). The alleviation of ALI by HDN occurs through protection of the intestinal mucosal barrier and reduction of LPS permeating in serum. The decrease in LPS inactivates the Nrf2/Keap1 signaling pathway and prevents inflammation.

**Conclusion:**

Hesperidin as a promising natural compound capable of modulating the gut–liver axis and strengthening endogenous antioxidant mechanisms, providing valuable insight into its potential therapeutic application in acute liver injury.

## Graphical abstract

**Figure:**
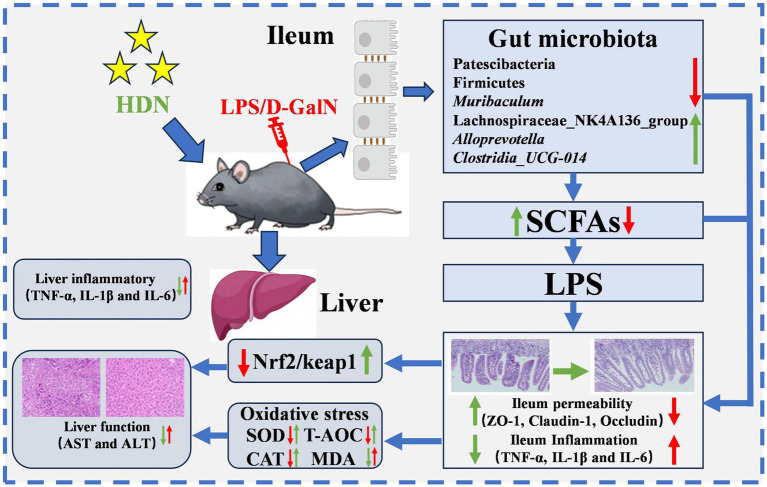

## 1 Introduction

An essential organ in the human body, the liver has a variety of roles in processes including metabolism and detoxification (Verma and Kaplowitz, 2009). Acute liver injury (ALI) brought on by poisons, or viruses has grown to be a major worldwide health issue (Lin et al., 2024; Nanizawa et al., 2024; Rutt et al., 2024; Wang F. et al., 2024). However, there are shortcomings in the current treatment approaches for ALI. Adverse effects are common with pharmacological medicines, such as corticosteroids and anti-tumor necrosis factor medications (Esparteiro et al., 2024; Vasconcelos et al., 2025). It is essential to find safe and efficient ALI preventive techniques. Numerous natural Chinese medications have clearly shown hepatoprotective qualities in clinical settings (Najafzadeh et al., 2010; Zhang et al., 2014).

Hesperidin (HDN), a flavonoid with hepatoprotective properties, is found in elevated amounts in citrus fruits (Hassanein et al., 2024; Wang X. et al., 2024). Numerous existing non-clinical investigations have shown that HDN has a range of bioactive effects, such as anti-tumor properties (Hassan et al., 2023; Lu et al., 2022), anti-inflammation (Wang J. et al., 2024), antioxidant (Panpan et al., 2023), and neuroprotective capabilities (Lai et al., 2022). With few adverse effects, it was predicted that the material in question had a high degree of therapeutic potential for treating either acute or chronic liver damage (Chen et al., 2024). Prior research has shown that HDN may prevent the production of nitric oxide and reactive oxygen species, providing protection against hepatotoxicity caused by lipopolysaccharides (Kaur et al., 2006). HDN’s antiapoptotic and anti-autophagic mechanisms, which shield the liver and kidneys from the harmful effects of sodium fluoride, are clearly responsible for its hepatoprotective and nephroprotective qualities (Caglayan et al., 2021). Nevertheless, it is still unclear exactly how HDN works in the setting of ALI.

The gut-liver axis is a two-way channel that allows the gut and its bacteria to continuously communicate with the liver (Albillos et al., 2020). A significant amount of data points to a clear correlation between the development and course of ALI and the makeup of the gut microbiota (Dumitru et al., 2024; Yang et al., 2024). The preservation of intestinal barrier homeostasis and alteration of the gut microbiota have been shown to be successful treatments for ALI (Li et al., 2024; Ohshima et al., 2025). The D-GalN/LPS liver damage model employed in the pre-laboratory phase was selected to further explore HDN’s mechanism of action in order to determine the material foundation for treating liver injury with HDN.

In this study, we determined the protective effect of HDN on acute liver injury in mice induced by D-GalN/LPS. The relationship between gut−liver axis impact and HDN intake was elucidated by testing the gut flora regulation, gut barrier function, and Nrf2/Keap1 pathway mechanisms.

## 2 Materials and methods

The Committee of Animal Care and Use at Zhejiang Provincial People’s Hospital, which gave its approval to the experiment’s design and methods (Approval No. 20250112212744). D-Galactosamine hydrochloride (D-GalN; ≥98% purity, Sigma-Aldrich, United States), lipopolysaccharide (LPS; *Escherichia coli* O111: B4, Sigma-Aldrich), hesperidin (HDN; ≥98%, Yuanye Biotechnology, China), and hesperidin derivative (HAD; ≥98%, Yuanye Biotechnology) were used in this study.

### 2.1 Experimental design and diets

**Figure 1:**
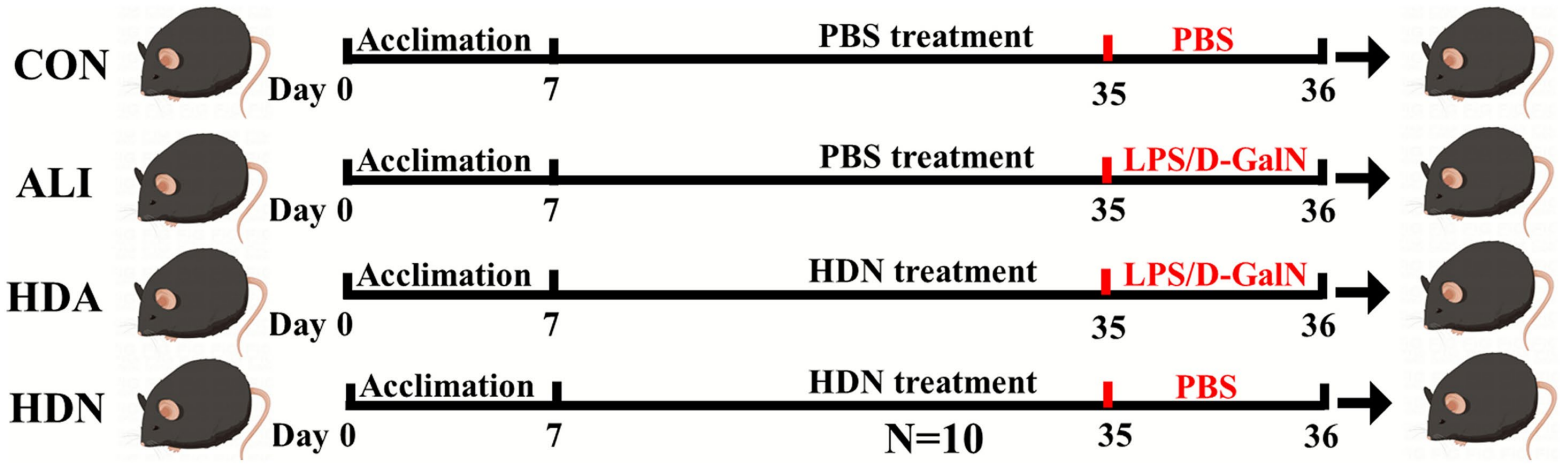
Experimental design.

A total of 40 healthy male C57BL/6 mice (18–22 g, 4 weeks old) were purchased from the Experimental Animal Center of Zhejiang Province (Hangzhou, China). C57BL/6 mice were housed in cages with a 12-h light/dark cycle, 23 ± 2 °C, and 55 ± 5% relative humidity. Mice were maintained on a standard chow diet (Beijing KeAo Biological Co., Ltd., China) containing 18% protein, 5% fat, and 5% fiber. In addition, the mice were divided into the control group (CON, the normal group, *n* = 10), the LPS&D-GalN group (ALI, the model group, *n* = 10), the HDN group (HDN, the positive drug group, feed 150 mg/kg HDN 28 days, *n* = 10), the HDA group (feed 150 mg/kg HDN 28 days HDN, *n* = 10). PBS (same volume) was used to create the CON and HDN groups. All groups except the CON and HDN group received intraperitoneal injections of 700 mg/kg D-GalN and 10 μg/kg LPS 28 days later. Blood samples were collected through retro-orbital plexus puncture under light isoflurane anesthesia. The samples were then centrifuged at 3,000 rpm for 10 min at 4 °C to obtain serum. Following anesthesia, which lasted six hours, tissue samples from the serum, ileum, and liver were harvested. Portions of the liver and ileum tissues were immediately fixed in formalin for further histopathological analysis (see Figure 1).

### 2.2 Histopathological analysis

The H&E staining was performed following previously published guidelines (Wu et al., 2018). To acquire images, the Olympus Microsystem (Tokyo, Japan) was used. The thickness of the tissue layer and the depth of septation in 15 appropriately oriented crypts, as well as the surrounding tissue layer area, were evaluated using image analysis software (Image-Pro Plus 6.0, Media Cybernetics, United States).

### 2.3 Biochemical assays

ELISA was used to measure the levels of serum TNF-α, IL-1β, IL-6, and IL-10 using assay kits (Nanjing Jiancheng, Bioengineering Institute, Nanjing, China). Using assay kits (Nanjing Jiancheng Bioengineering Institute, Nanjing, China), the activity of glutathione peroxidase (GSH-Px), catalase (CAT), and total antioxidant capacity (T-AOC) and MDA were measured on a SpectraMax M5 apparatus (Molecular Devices, United States) in accordance with the manufacturer’s instructions.

### 2.4 Reverse transcription polymerase chain reaction

The concentration and purity of total RNA were assessed using a nanodrop spectrophotometer (ND-2000, Thermo Fisher Scientific), and RNA was extracted using the RNAiso Plus Kit (Takara, Japan). Quantitative reverse transcription polymerase chain reaction (RT-PCR) analysis was performed using the ABI 7500 fluorescent detection equipment and SYBR green (Takara, Japan). Table 1 shows the primer sequences for samples that were assayed in triplicate. The relative gene expression was calculated using the 2^−ΔΔCT^ method, with β-actin acting as an internal reference gene.

**Table 1:** Sequences of the oligonucleotide primers used for quantitative real-time PCR.

Gene name	Accession number	Sequence (5′ → 3′)
*β-actin*	NM_007393.5	F: ATTCATGCGGCCAAAGAGGA
		R: CCGTGCGAGTCAGAGAAAGA
*TNF-α*	NM_001278601.1	F: GCGGGGTAAGGTCAGTTTGT
		R: TTTCGGACTCAGCATCACCG
*IL-1β*	XM_006498795.5	F: GAGTCGGATGGTTCTTCCCC
		R: ACAGGAGGTTGGCATGTTGG
*IL-6*	NM_001314054.1	F: TGACGGCATTTGAAGGGGTC
		R: GCAGCGGTCTGAAGGTTTGA
*IL-18*	NM_001357221.1	F: TAACGCAGAACAACGGCAAC
		R: ATGACCGGCCTTTTCTTGGT
*IL-10*	XM_036162094.1	F: GTCCCTGCTGGAGGACCTTA
		R: GATGGCTGGACTGTGGTTCT
*IL-22*	XM_006513865.4	F: CCCAGAGTCAACGCTAACCA
		R: TCGAGGATGTTGGCATGGAG
*Claudin-1*	NM_016674.4	F: TGACCTTCCCACACAATCCC
		R: AACAGCACGCAGCTACTCAA
*Claudin-2*	NM_016675.5	F: GGAGAGGCCCTTTACTTGGG
		R: GACTCCAGCTACCAGTGAGA
*Claudin-5*	NM_013805.4	F: CCCTGCGAACTGCTTTTTGT
		R: GTGCAATCCCGTCATGCTTC
*Occludin*	NM_001360537.1	F: AGAGCATAGATGTGTCGGCG
		R: TCACGAATGGCAATGACCCA
*ZO-1*	NM_001163574.2	F: TCAGAGCGTCACACCAGAAC
		R: CGTCATACCGACTTGGAGGG
*Keap1*	NM_001110305.1	F: TGAGCCCTCGTAGGGTGG
		R: CAGTTCCTCTGCTAGCCGTT
*Nrf2*	NM_001399226.1	F: AACTACCTGCCAATCCAGGC
		R: GCCTCGTTGAACTGCTCCTT
*HO1*	NM_010442.2	F: ATGGGACGGCGATCTTTCTC
		R: AACGAGGAAACAAAGCGCAA
*NQO1*	NM_008706.5	F: ACTCTTGCTTCGCCTGTTCT
		R: TCGGAAGGTATCGGTGTGTG

### 2.5 Western blotting

The BCA technique was used to ascertain the protein concentrations in ileum lysates, and Sigma (Saint Louis, MO, United States) provided the lysis buffer. To extract the cellular supernatants, the lysates were homogenized and centrifuged for 10 min at 4 °C at 12,000 g. The protein samples were electrotransferred onto PVDF (polyvinylidene difluoride) membranes (Millipore, MA, United States) after being separated using 12% SDS-PAGE. Protein-containing membranes were blocked for 2 h at room temperature using 5% dry skim milk, and then they were incubated with primary antibodies Nrf2, and HO1 (Abcam, Cambridge, United Kingdom), as well as β-actin (Cell Signaling Technology, MA, United States), for an entire night at 4 °C. After five TBST rinses, the membranes were incubated with a secondary antibody labeled with horseradish peroxidase (HRP) for 2 h at 26 °C. After that, protein bands were found using the Millipore chemiluminescent HRP substrate kit (MA, United States) and an imaging system (Tanon, China). Band intensity data acquired using ImageJ software were used to adjust the protein expression level to β-actin.

### 2.6 Gut microbial DNA extraction and high-throughput sequencing

Using the TIANamp Stool DNA Kit (Tiangen, Beijing, China), microbial genomic DNA was extracted from the cecum contents of mice. The V3 to V4 region of the 16S rRNA gene was amplified using the 341F/805R primer pairs, and sequencing was performed on an Illumina MiSeq platform (Illumina Inc., San Diego, CA, United States). The QIIME program (version 1.9.1) clustered input sequences with 97% similarity into operational taxonomy units (OTU). Alpha diversity, containing Shannon, Simpson, Ace, and Chao were calculated to reflect the bacterial diversity and richness. On unweighted UniFrac, OTU-level beta diversity was calculated. Utilize UniFrac-based principal coordinate analysis (PCoA) to acquire principal coordinates and display complex data. Using non-metric multidimensional scaling (NMDS), differences in community structure between samples were calculated. The relative abundance of significant differences at the phylum, genus and OTU levels were calculated. The NCBI SRA accession number: PRJNA1366528.

### 2.7 Detection of SCFA levels

The following conditions were used to measure the levels of SCFA: a flow rate of 2 mL/min, an initial column temperature of 90 °C, a duration of 6 min, a rate of 10 °C/min to 200 °C, and another 6 min. The gas chromatograph used was an Agilent 7890 from Palo Alto, CA, United States. There is a 2 mL/min flow rate for the carrier gas. With a hydrogen flow rate of 30 mL/min and an air flow rate of 300 mL/min, the FID detector is operating at 250 °C.

### 2.8 Statistical analysis

SPSS (version 20.0; IBM Inc., NY, United States) statistical software was used to perform a one-way analysis of variance (ANOVA) followed by multiple comparison tests using the Turkey’s approach for data detection. Means were reported with their standard deviation (SD), and a significance level of *p* < 0.05 was used to indicate a statistically significant difference. The graphing software used was GraphPad Prism 8.4.2.

## 3 Results

### 3.1 HDN ameliorated LPS&D-GalN induced hepatic histological in mouse

**Figure 2:**
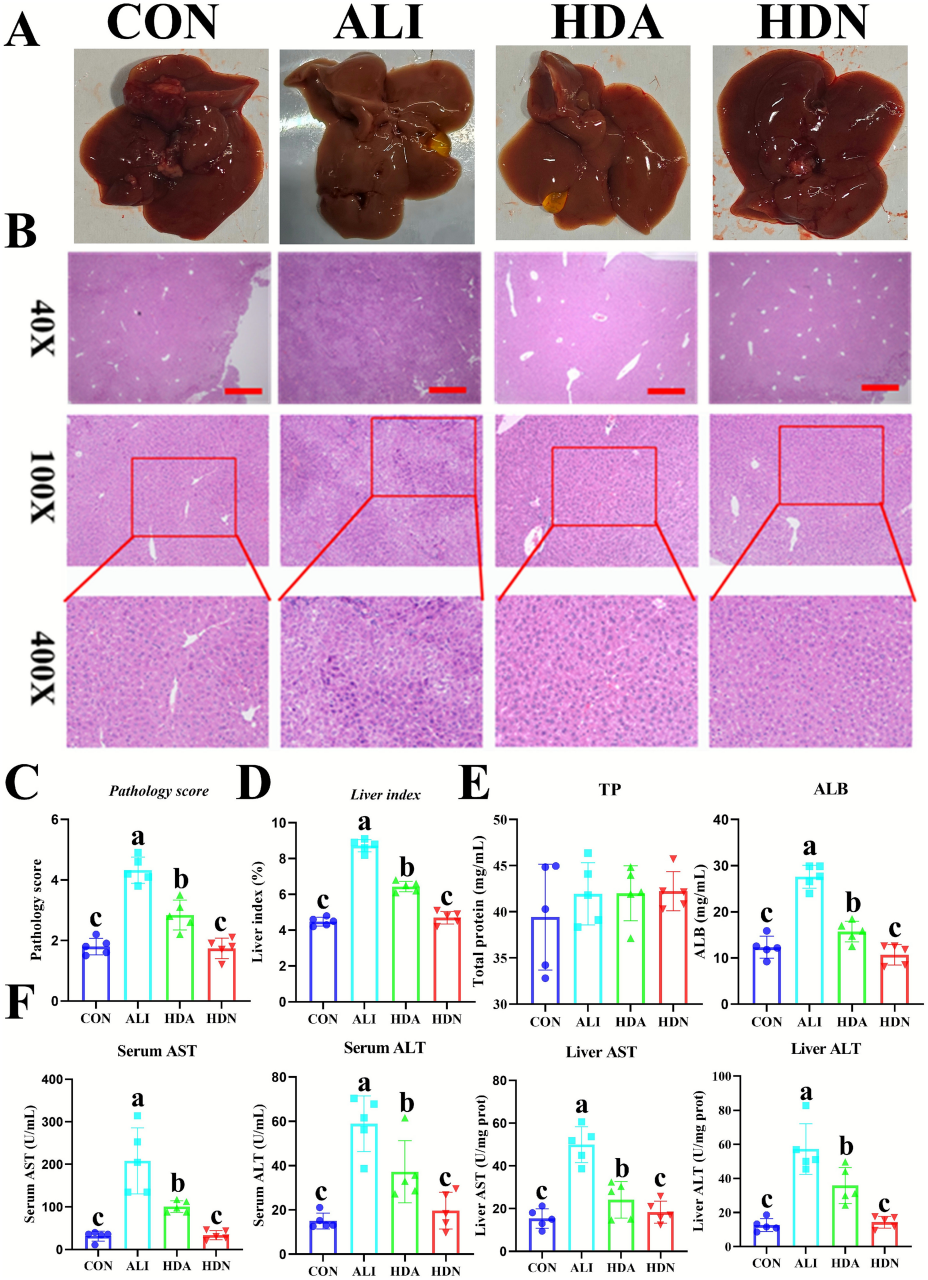
HDN administration relieves liver injury induced by LPS&D-GalN. **(A)** Liver morphology. **(B)** Representative H&E-stained histologic sections of the liver (scale bars: 200 μm). **(C)** Pathology score. **(D)** Liver index. **(E)** TP and ALB content in serum. **(F)** AST and ALT in serum and liver. Data are presented as means ± SD (*n* = 5). ^a,b,c^Means within a row with different superscripts differ significantly (*p* < 0.05).

The CON group did not exhibit any discernible liver histological alterations or abnormalities. A consistent pattern was seen in the alignment of the core veins of the hepatic lobules, and the cell morphology was found to be normal. On the contrary, after LPS&D-GalN injection, there were visible histological alterations such as abnormally distributed hepatocytes, irregular cell shape, inflammatory cell infiltration, and a significant proportion of ruptured hepatic cells. The HDA group exhibited superior liver gross architecture and reduced inflammatory cell infiltration compared to the ALI group (Figures 2A–D). LPS&D-GalN injection also significantly increased the ALB content in serum, while HDA reversed this tendency (Figure 2E). In addition, the LPS&D-GalN injection resulted in a significant increase (*p* < 0.05) in the content of ALB in serum, and activity of ALT and AST in serum and liver, indicating that LPS&D-GalN caused ALI. However, the HDN administration significantly (*p* < 0.05) decreased the ALT and AST levels (Figure 2F). The above results suggest that HDN has the potential to protect the liver from the LPS challenges.

### 3.2 HDN ameliorated LPS&D-GalN induced intestinal physical barrier function injury in mouse

**Figure 3:**
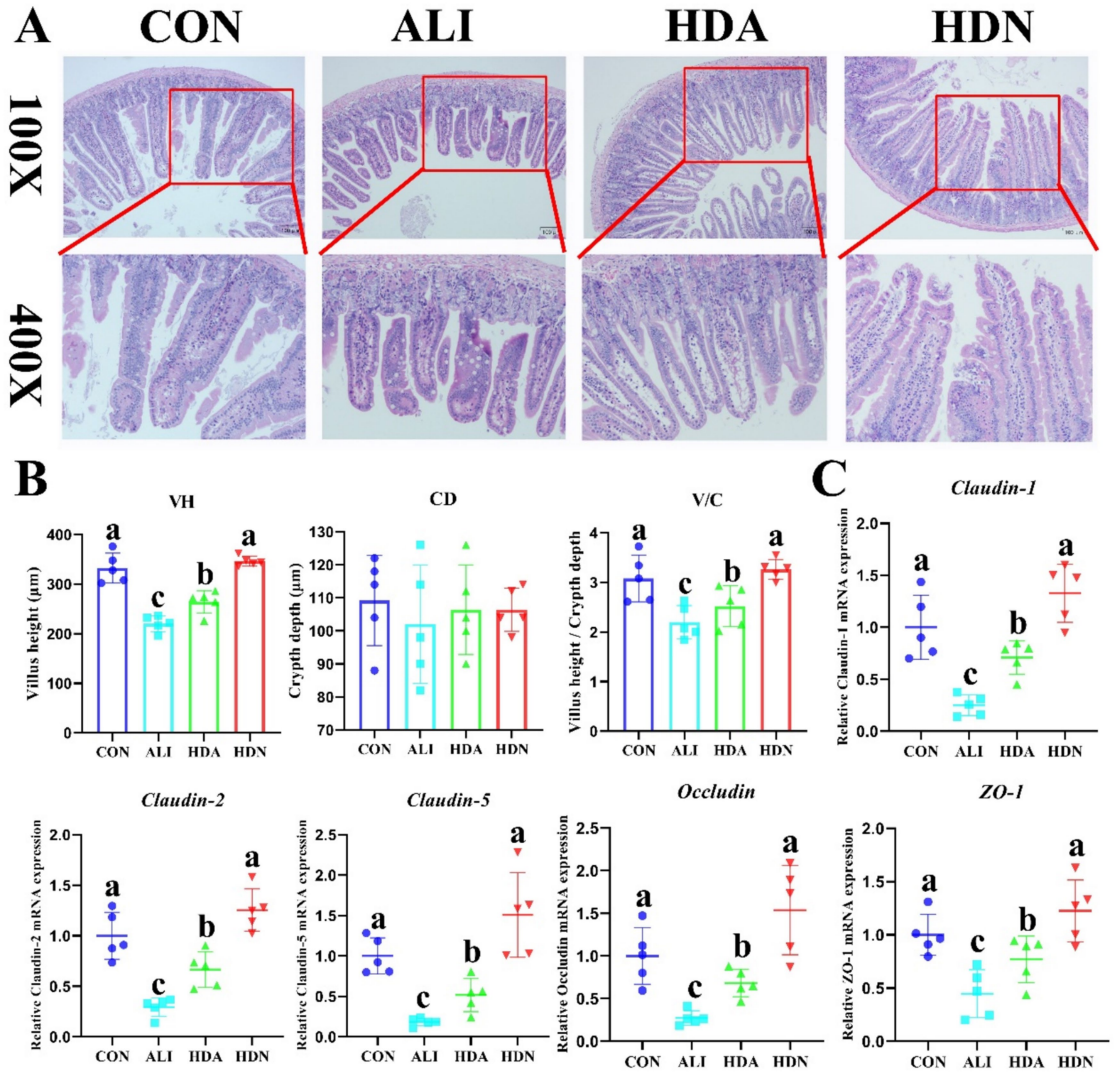
HDN enhanced the intestinal physical barrier function in ALI mouse. **(A,B)** Histomorphology of the ileum in mice (scale bar: 100 μm). **(C)** *Claudin-1*, *Claudin-2*, *Claudin-5*, *Occludin*, and *ZO-1* gene expression in ileum. Mean ± SD (*n* = 5). ^a,b,c^Means within a row with different superscripts differ significantly (*p* < 0.05).

Figures 3A,B shows that ileum villus height and villus height/crypt depth ratio (VCR) were all significantly (*p* < 0.05) reduced in LPS&D-GalN-challenged mouse compared to the CON group. Supplementation with HDN increased villus height and VCR in LPS&D-GalN-challenged mouse (*p* < 0.05). In addition, we also found that ileum *Claudin-1*, *Claudin-2*, *Claudin-5*, *Occludin*, and *ZO-1* gene expression was significantly (*p* < 0.05) reduced by LPS&D-GalN administration compared to the CON group, while supplementation with HDN reversed this trend (Figure 3C). Our findings suggest that the administration of HDN has the potential to improve the integrity of the intestinal physical barrier in ALI mouse.

### 3.3 HDN supplementation suppressed LPS&D-GalN-induced inflammatory responses

**Figure 4:**
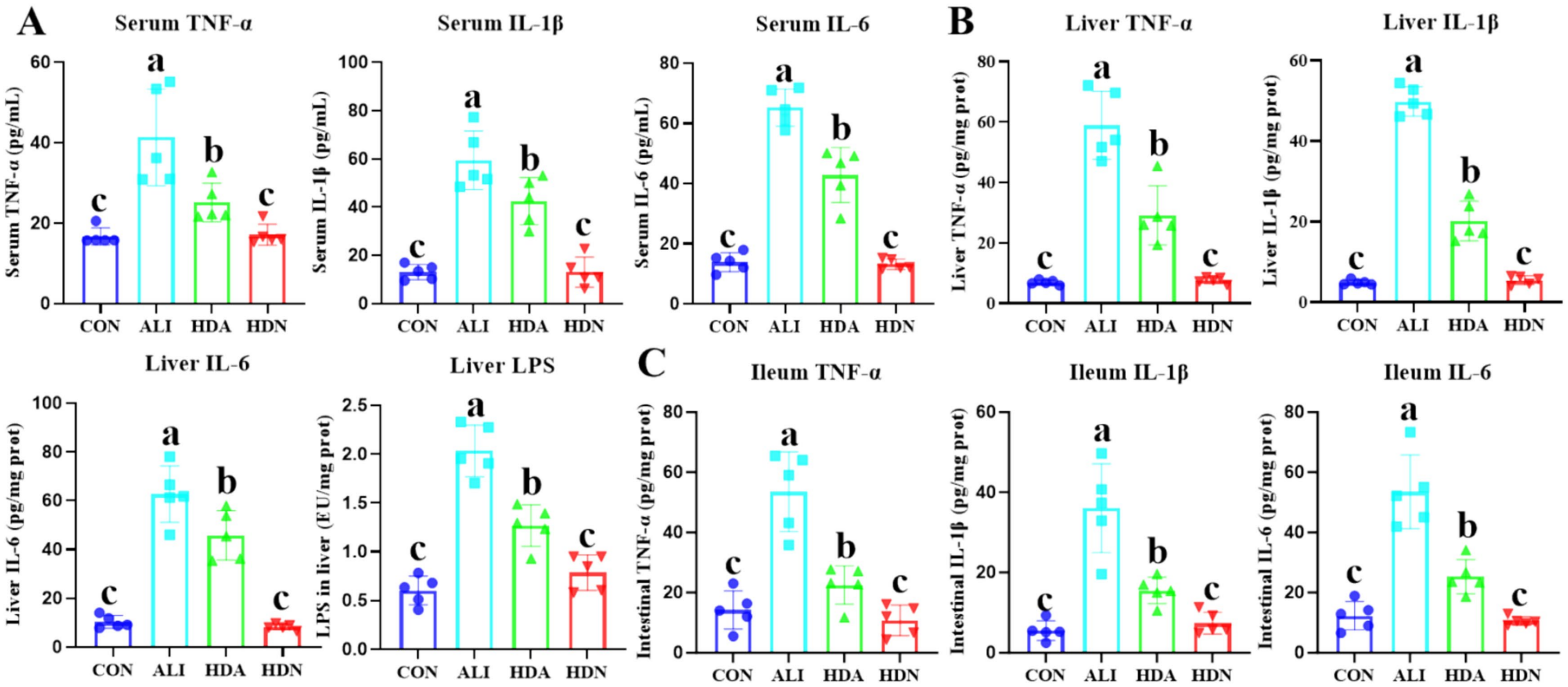
HDN decreased the inflammatory cytokines content in ALI mouse. **(A)** TNF-α, IL-1β, and IL-6 content in serum. **(B)** TNF-α, IL-1β, IL-6, and LPS content in liver. **(C)** TNF-α, IL-1β, and IL-6 content in ileum. Mean ± SD (*n* = 5). ^a,b,c^Means within a row with different superscripts differ significantly (*p* < 0.05).

**Figure 5:**
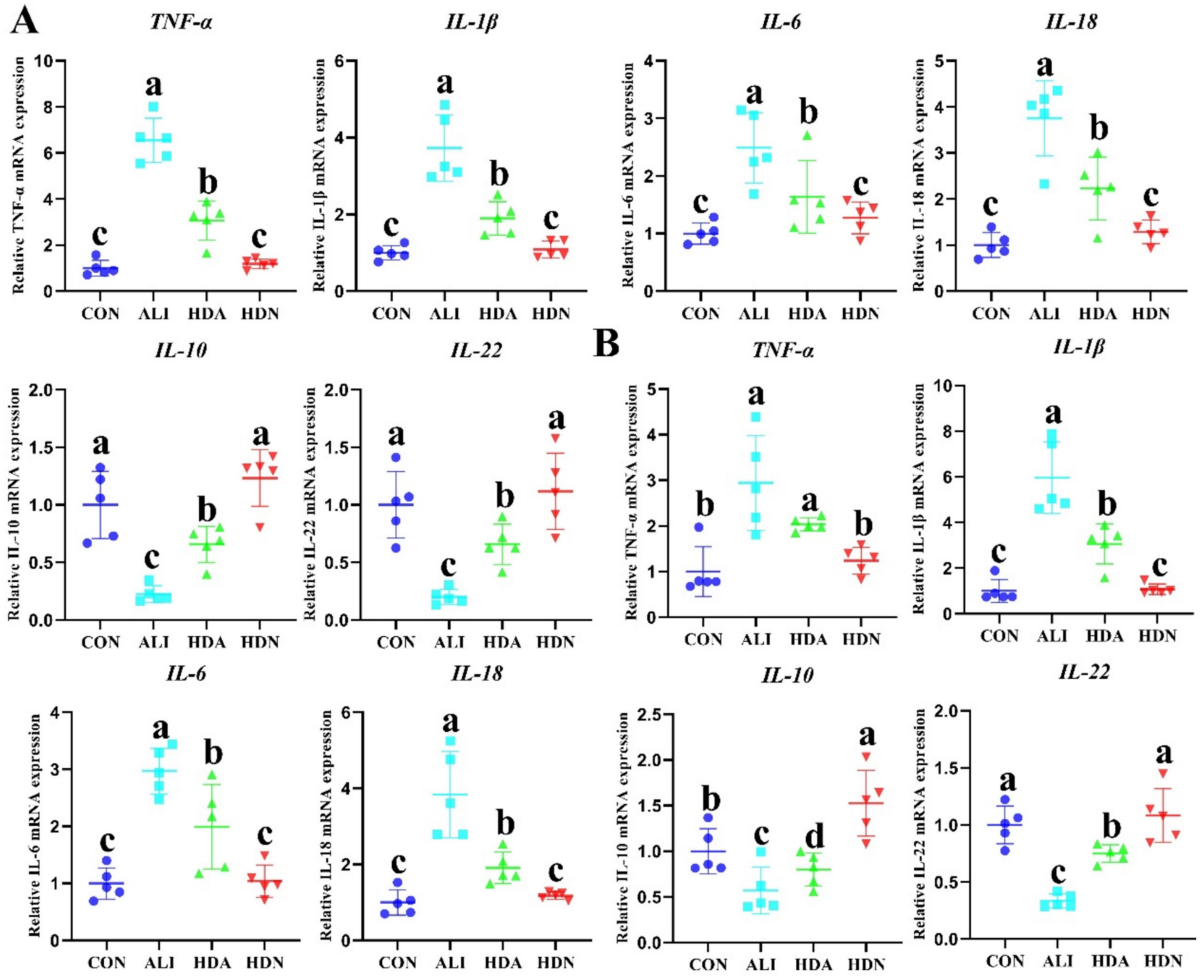
HDN decreased the inflammatory cytokines gene expression in ileum **(A)** and liver **(B)** of ALI mouse. Mean ± SD (*n* = 5). ^a,b,c^Means within a row with different superscripts differ significantly (*p* < 0.05).

The release of damage-associated molecular patterns by deceased hepatocytes leads to a secondary insult in ALI, triggering an immunological response that exacerbates liver damage. Thus, inflammatory damage is the primary pathological mechanism in ALI. ELISA results demonstrated that LPS&D-GalN significantly (*p* < 0.05) increased the TNF-α, IL-1β and IL-6 level in serum, liver and ileum, showing that LPS&D-GalN induced these inflammatory mediators, whereas the HDN inhibited the production of the inflammatory mediators (Figures 4A,B). The qPCR results also demonstrated that LPS&D-GalN markedly (*p* < 0.05) up-regulated the mRNA expression of *TNF-α*, *IL-1β*, *IL-6*, and *IL-18*, and downregulated *IL-10* and *IL-22* gene expression compared with CON group, which were reversed by HDN supplementation in ileum and liver (Figure 5).

### 3.4 HDN supplementation suppressed LPS&D-GalN-induced hepatic oxidative stress

**Figure 6:**
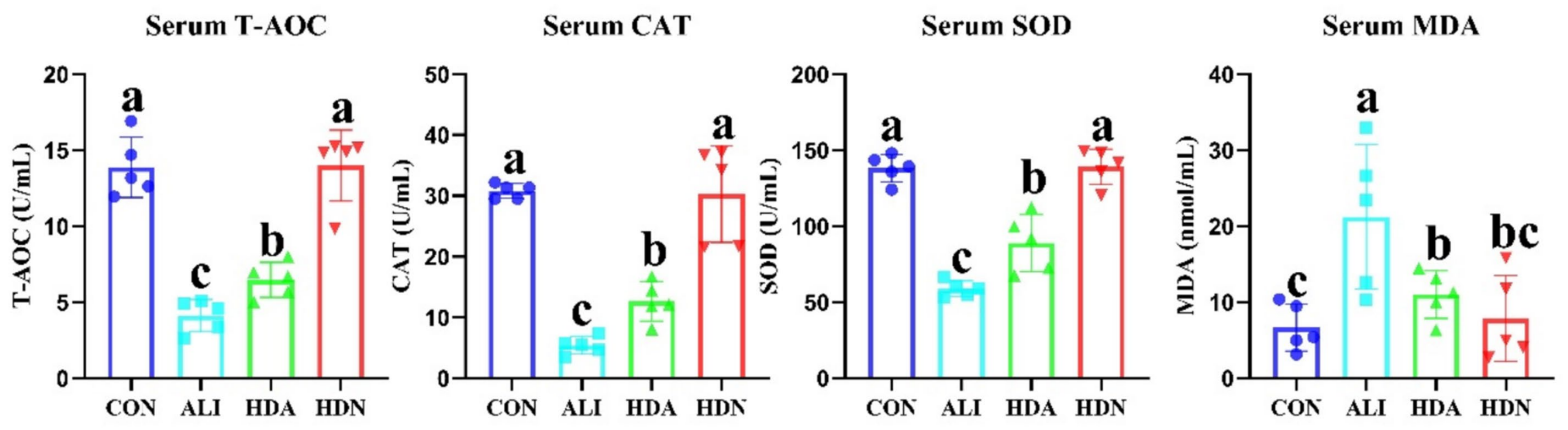
HDN enhanced serum antioxidant status in ALI mouse. Mean ± SD (*n* = 5). ^a,b,c^Means within a row with different superscripts differ significantly (*p* < 0.05).

Compared with CON group, LPS&D-GalN significantly (*p* < 0.05) decreased the serum antioxidant status (T-AOC, CAT, and SOD), while HDN supplementation reversed this trend. In addition, serum MDA levels were significantly (*p* < 0.05) lower in the CON and HDA groups than that in the ALI group (Figure 6).

### 3.5 Gut microbiota analysis

#### 3.5.1 Microbiota diversity in intestinal contents

**Figure 7:**
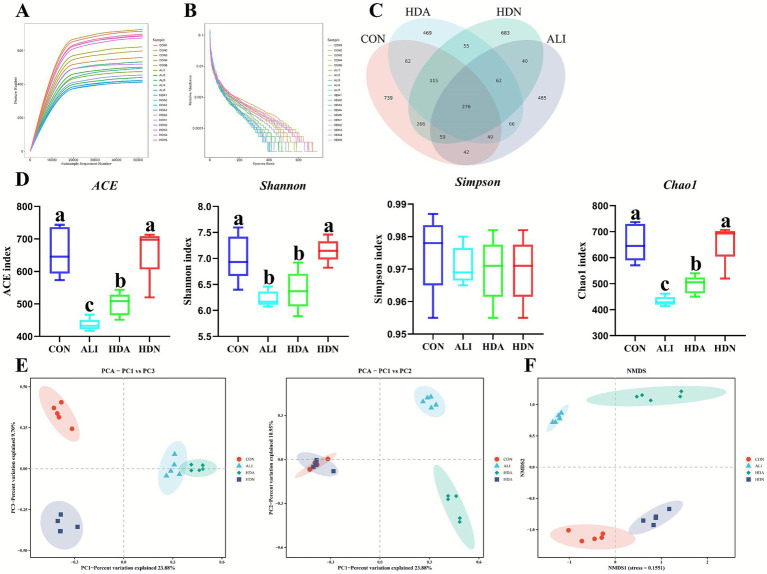
Effect of HDN on gut microbiota diversities of ALI mice. **(A,B)** Species cumulative curve. **(C)** Venn diagram. **(D)** ACE, Shannon, Simpson and Chao index. **(E)** PCA of weighted UniFrac distance. **(F)** NMDS analysis of weighted UniFrac distance. Mean ± SD (*n* = 5). ^a,b,c^Means within a row with different superscripts differ significantly (*p* < 0.05).

The curves revealed that OTU richness per sample was nearing saturation, suggesting adequate sequencing depth (Figures 7A,B). Venn chart indicated that the HDA group comprised 469 distinct OTUs compared with other groups (Figure 7C). Alpha diversity analysis (Figure 7D) demonstrated that the ALI stimulation significantly (*p* < 0.05) decreased the alpha diversity index (Feature, ACE, Chao, and Shannon), while HDA reversed this tendency. In addition, the PCA and NMDS plots of cecum microbiota (Figures 7E,F) confirmed that there were obviously (*p* < 0.05) changes in the microorganism communities among all groups. This finding demonstrated that HDN affected the enteric microorganism community structure within the intestine.

#### 3.5.2 Cluster analysis

**Figure 8:**
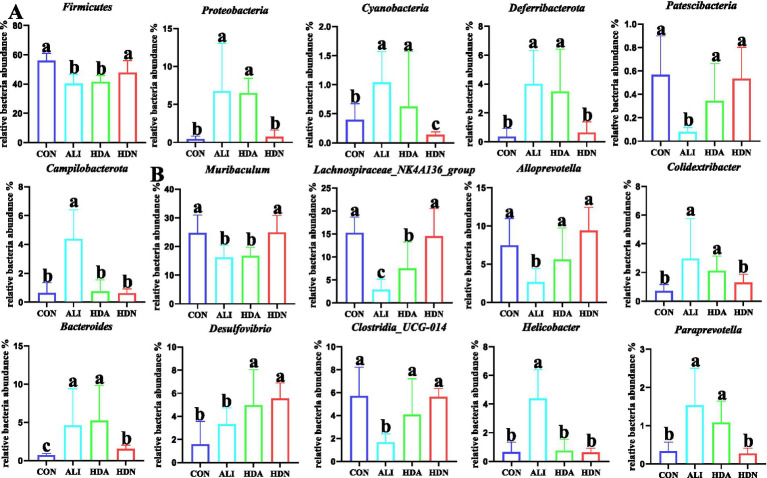
Bacterial taxonomic composition of cecum contents. **(A,B)** Relative abundance at the phylum level and genus level. Mean ± SD (*n* = 5). ^a,b,c^Means within a row with different superscripts differ significantly (*p* < 0.05).

At phylum level, we found that compared with CON group, ALI significantly (*p* < 0.05) decreased the relative abundance of Firmicutes and Patescibacteria, but increased the relative abundance of Proteobacteria, Cyanobactertia, Deferribacterota and Campilobacterota (Figure 8A). There were no significant (*p* > 0.05) differences in the relative abundance of Firmicutes, Patescibacteria, Proteobacteria, Cyanobactertia, Deferribacterota and Campilobacterota between ALI and HDA groups. As shown in Figure 8B, at genus level, compared with CON group, ALI significantly (*p* < 0.05) decreased the relative abundance of *Muribaculum*, Lachnospiraceae-NK4A136-group, *Alloprevotella* and *Clostridia-UCG-014*, and increased the *Colidextribacter*, *Bacteroides*, *Desulfovibrio*, *Helicobacter* and *Paraprevotella*. In addition, compared with ALI group, HDA significantly (*p* < 0.05) increased the relative abundance of Lachnospiraceae-NK4A136-group, *Alloprevotella*, *Desulfovibrio* and *Clostridia-UCG-014*, and decreased the relative abundance of *Colidextribacter*, *Helicobacter* and *Paraprevotella*.

#### 3.5.3 Overall structure modulation of gut microbiota

**Figure 9:**
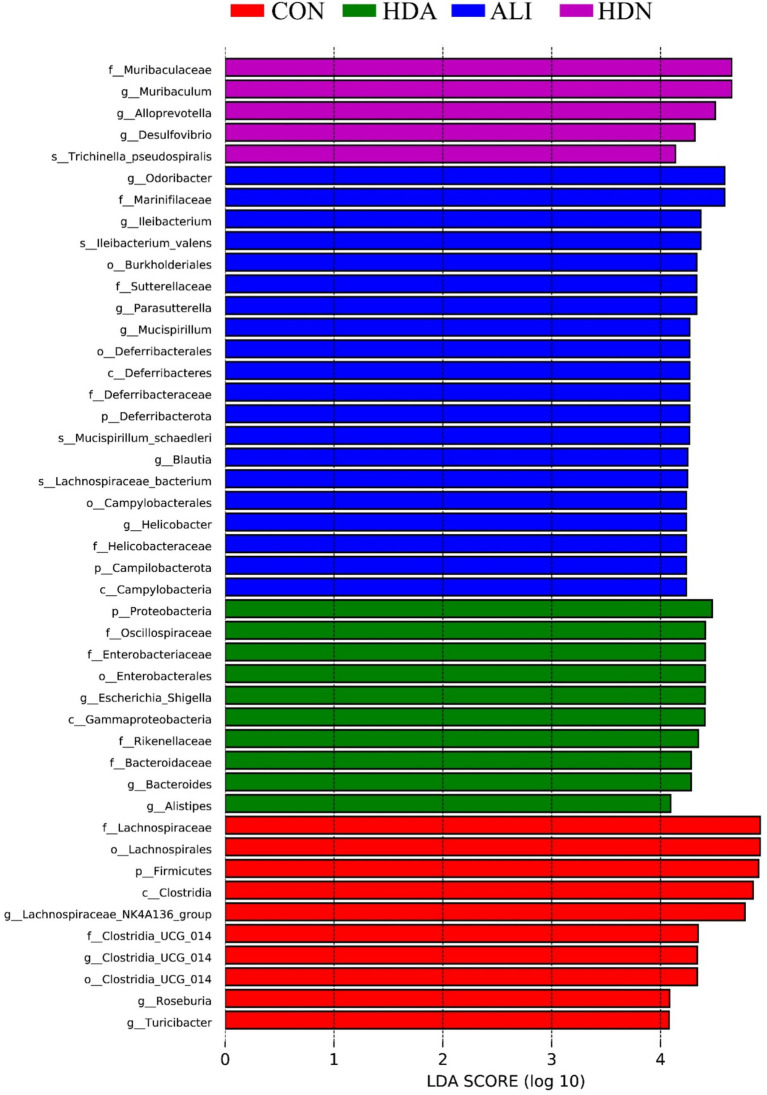
LEfSe bar.

Consistent with the bacterial changes described above, the cladogram generated from the linear discriminant analysis effect size (LEfSe) analysis, showed different intestinal microbiota compositions in all groups of mouse (Figure 9). The comparison of dominant bacterial taxa at the genus level suggested that ALI increased the relative abundance of *Odoribacteria*, *Ileibacterium*, *Parasutterella*, *Mucispirillum*, *Blautia* and *Helicobacter* compared with CON group. In addition, compared with ALI group, HDA significantly (*p* < 0.05) enhanced the relative abundance of *Escherichia-Shigella*, *Bacteroides* and *Alistipes*.

#### 3.5.4 Functional metagenomics prediction of gut microbiota

**Figure 10:**
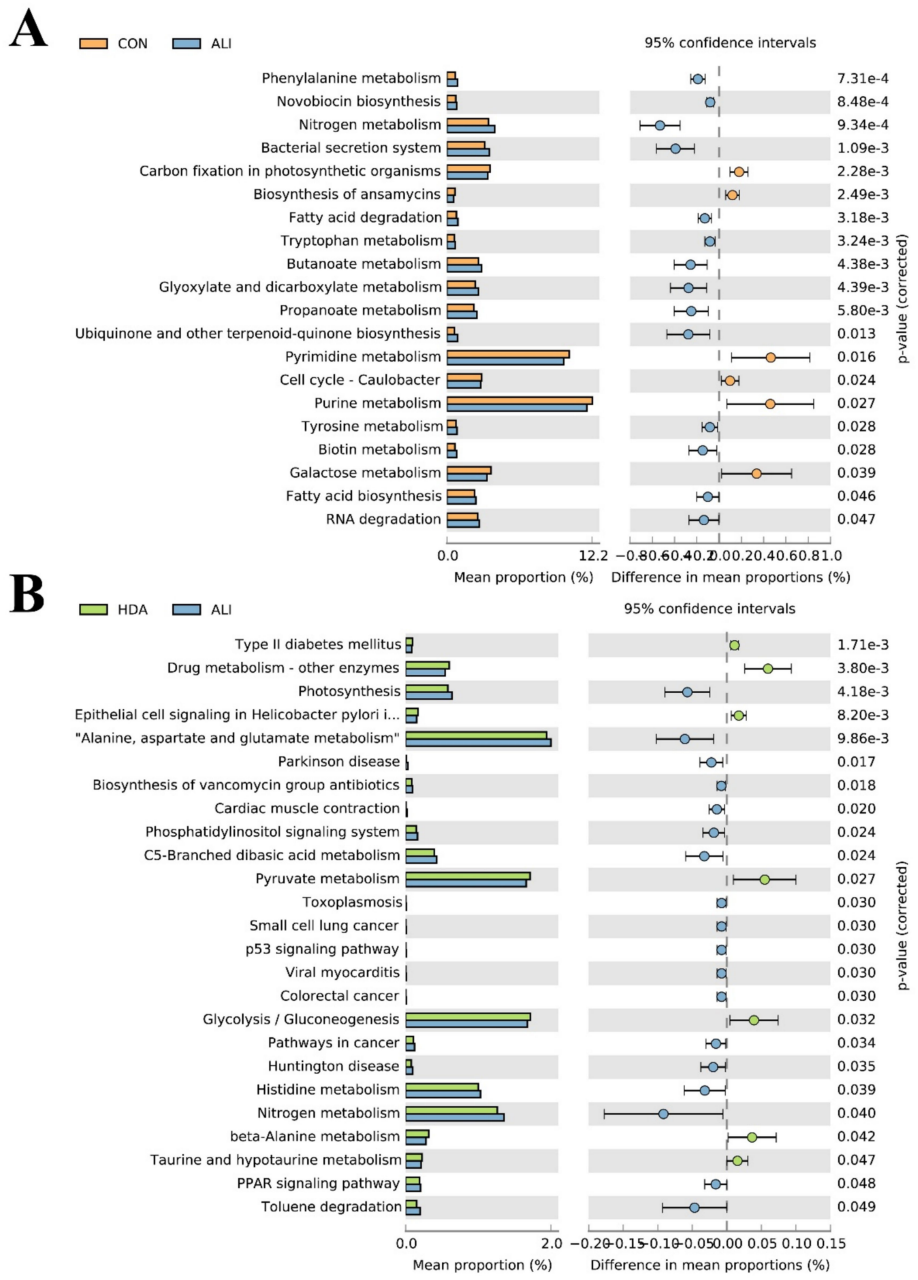
Comparison to predicted metabolic pathway abundance between the groups by statistical analysis of taxonomic and functional profiles (STAMP) at Level 3. **(A)** CON vs. ALI. **(B)** HDA vs. ALI.

To determine whether HDN-induced microbial changes modulate the metabolic function of gut microbiota, 16S rRNA gene sequencing-based functional metagenomics prediction of gut microbiota was performed (Figure 10A). Our results showed that 20 pathways at the third level of KEGG pathway were significantly (*p* < 0.05) altered following by HDN treatment, including significantly increased proportions of tryptophan metabolism, fatty acid degradation, butanoate metabolism, tyrosine metabolism and fatty acid biosynthesis (Figure 10B). In addition, we also found that HDN supplementation significantly increased the abundance of Pyruvate metabolism, beta-Alanine metabolism.

#### 3.5.5 HDN reversed the reduction of SCFAs in the cecum of LPS&D-GalN-challenged mouse

**Figure 11:**
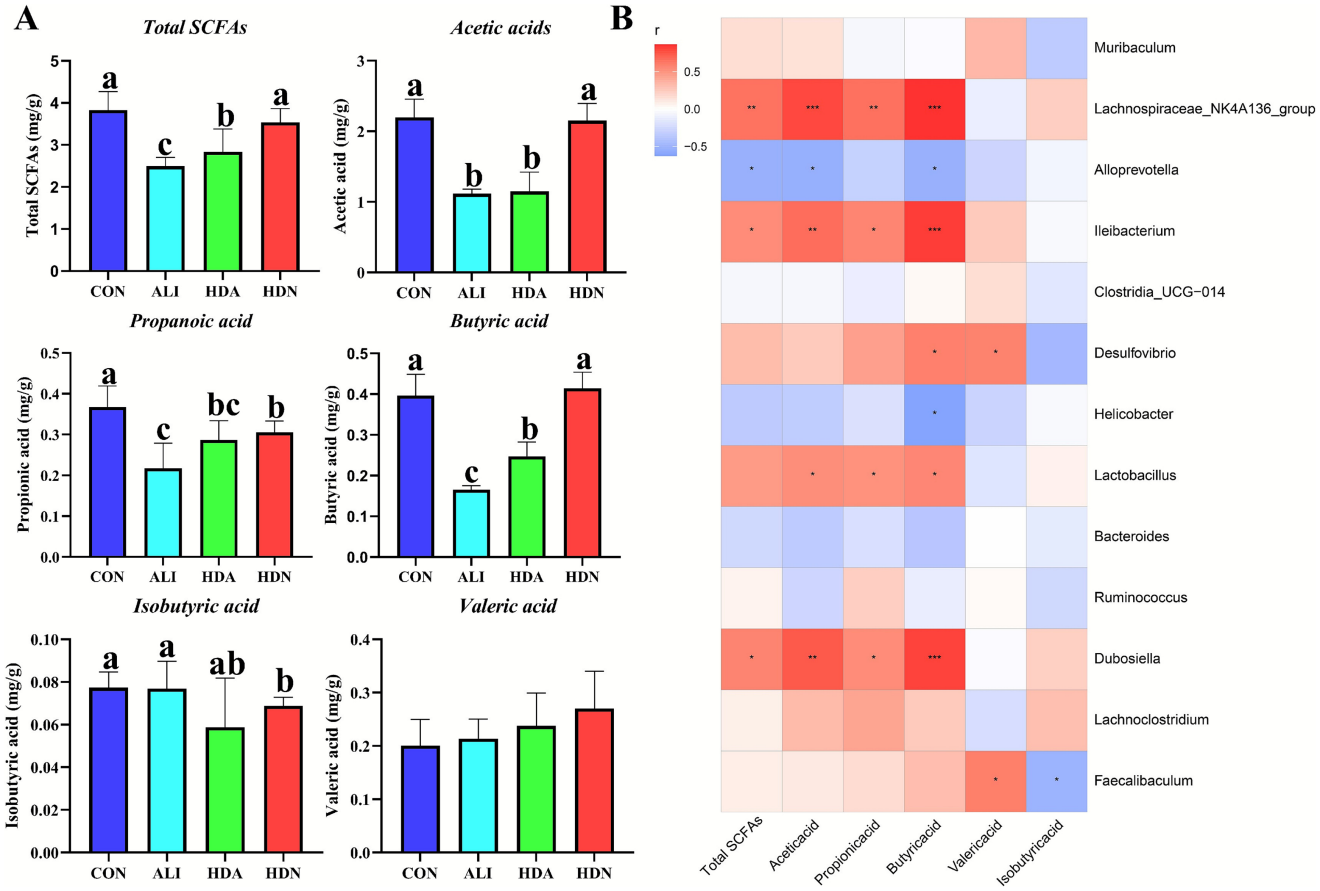
Effect of HDN on the content of SCFAs in cecal contents of LPS&D-GalN-challenged mouse mice. **(A)** SCFAs content in cecum. **(B)** Correlation analysis between bacterial community and SCFAs. Mean ± SD (*n* = 5). ^a,b,c^Means within a row with different superscripts differ significantly (*p* < 0.05).

The ALI group exhibited a substantial decrease in the total short-chain fatty acids (SCFAs), acetic acid, propionic acid, and butyric acid contents of the cecum. Conversely, the HDA treatment effectively restored the contents to their normal levels (Figure 11A). The correlation analysis between bacterial community and SCFAs demonstrated that Lachnospiraceae-NK4A136-group, *Ileibacterium*, *Desulfovibrio*, *Lactobacillus* and *Dubosiella* were positively associated with total SCFAs, acetic acid, propionic acid and butyric acid content, while *Alloprevotella* were negatively associated with total SCFAs, acetic acid and butyric acid (Figure 11B).

### 3.6 HDN activated the Nrf2/Keap1 signaling pathway in LPS&D-GalN-challenged mouse

**Figure 12:**
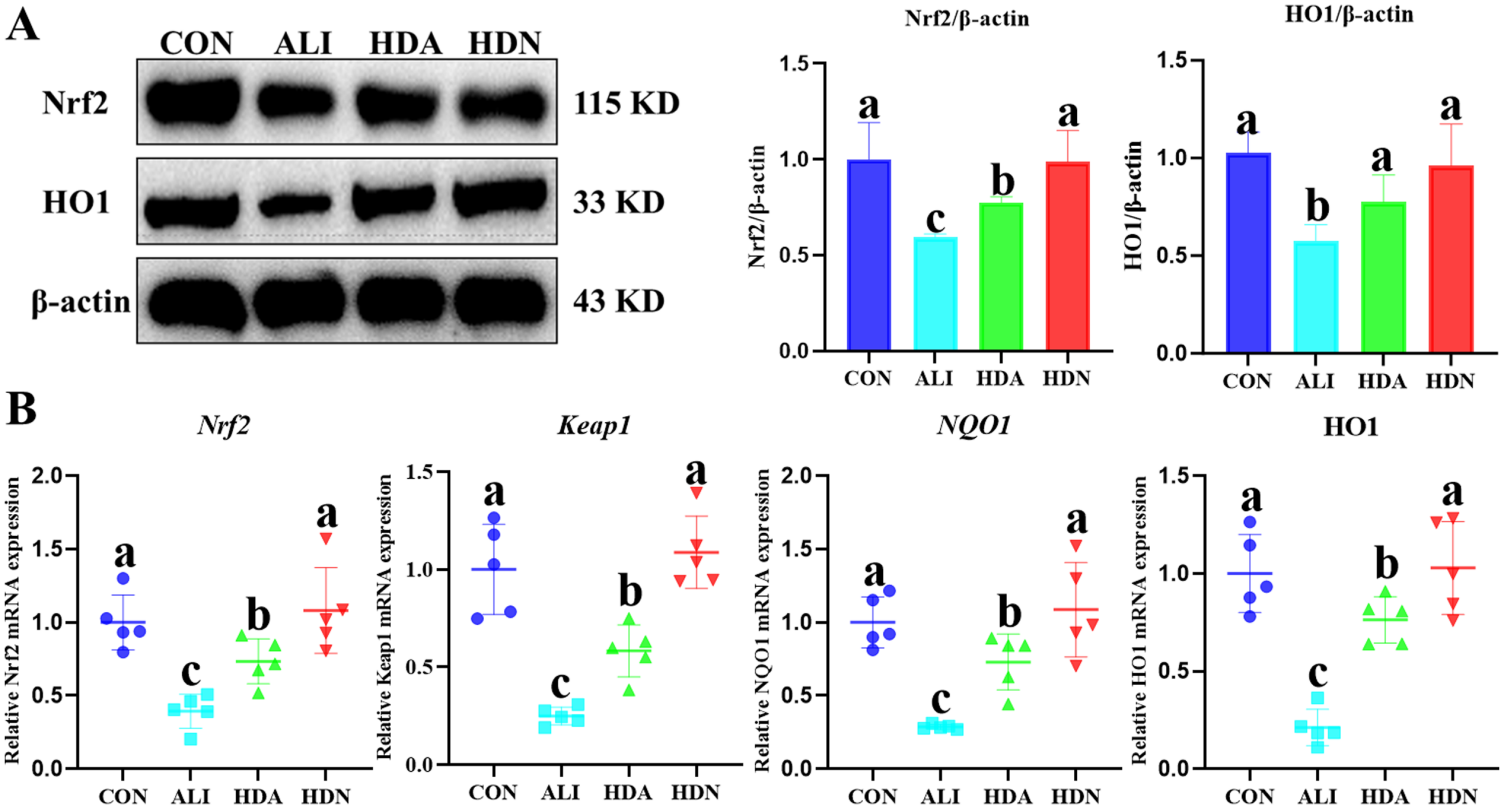
Effects of dietary supplementation with HDN on Nrf2/keap1 signal pathways related gene expressions. **(A)** The western blot results; **(B)** qPCR results. Mean ± SD (*n* = 5). ^a,b,c^Means within a row with different superscripts differ significantly (*p* < 0.05).

The results of the previous flora analyses indicated that feeding HDN significantly altered the abundance of tryptophan metabolism-related microbiota, and tryptophan metabolism is closely related to the Nrf2/Keap1 signaling pathway, so we studied the expression of key genes in the Nrf2/Keap1 signaling pathway. The western blot results demonstrated that ALI significantly (*p* < 0.05) decreased the protein levels of Nrf2 and HO1 of ileum compared with the CON group, while HDN supplementation reversed this tendency (Figure 12A). In addition, qPCR results also demonstrated that ALI significantly (*p* < 0.05) decreased the gene expression levels of *keap1*, *Nrf2*, *NQO1* and *HO1* compared with CON group in ileum, while HDN supplementation reversed this tendency (Figure 12B).

### 3.7 Correlation heat map

**Figure 13:**
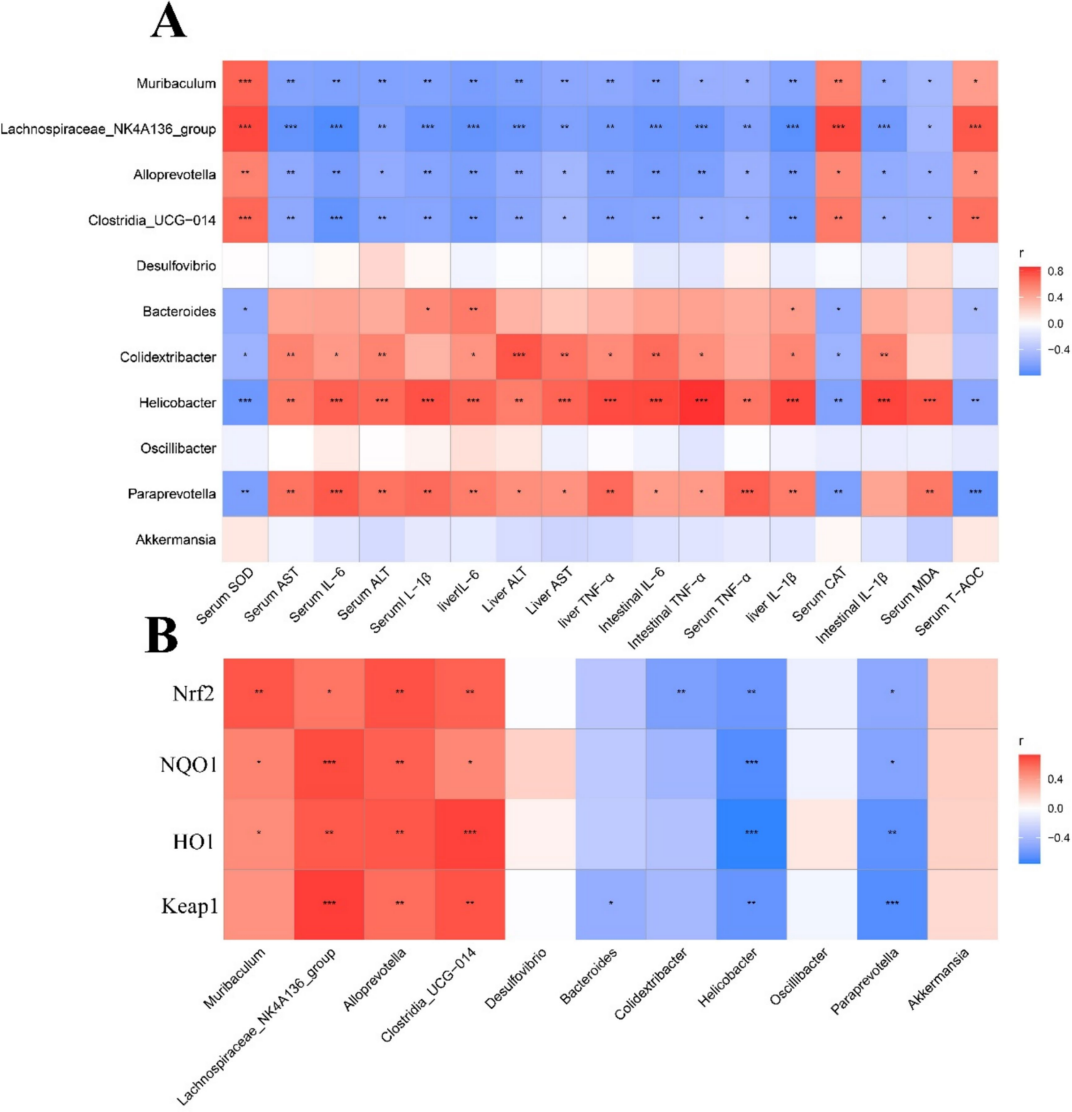
Pearson’s correlation analysis. Correlation of phenotypic variables and microbial communities **(A)** and Nrf2/Keap1 signal pathway related genes **(B)**. The color and the dot size represent the correlation coefficient. ^*^p < 0.05, ^**^p < 0.01, and ^***^p < 0.001.

Perform Spearman correlation analysis to further investigate specific relationships between phenotypic variables and the functional composition of microbial communities or microbial metabolism (Figure 13). As shown in Figure 13A, we found that inflammatory factors (TNF-α, IL-6 and IL-1β), and oxidative stress factor (MDA) were positively with the *Colidextribacter*, *Helicobacter*, and *Paraprevotella*, and negatively with *Lachnospiraceae-NK4A136-group*, *Muribaculum*, *Alloprevotella*, and *Clostriadia-UCG-014*. In addition, we also found that *Nrf2*, *NQO1*, *HO1* and *keap1* gene expression levels were positively with the Lachnospiraceae-NK4A136-group, *Muribaculum*, *Alloprevotella*, and *Clostriadia-UCG-014*, and negatively with *Colidextribacter*, *Helicobacter*, and *Paraprevotella* (Figure 13B).

## 4 Discussion

The liver plays a significant role in detoxification and metabolic balance, making liver disease a well-known problem (Johnson et al., 2015). There is growing evidence from recent studies that HDN may be used therapeutically to prevent and treat acute liver damage (ALI). By controlling the gut microbiota to activate the Nrf2/Keap1 signaling pathway, we showed in this research that HDN can greatly decrease inflammation, oxidative stress, and acute liver damage.

When hepatocytes are injured, the integrity of the cell membrane is compromised, releasing transaminases (ALT and AST) into the circulation, which raises serum ALT and AST activity. AST and ALT are thus well acknowledged as essential biochemical indicators used to determine the severity of liver lesions (Geuken et al., 2004). Our findings demonstrated that mice treated with LPS&D-GalN had considerably higher serum AST and ALT levels and severe liver damage when compared to the control group. HDN, however, reverses these biochemical markers. Prior research has shown that HDN reduces the levels of these chemicals in the blood, hence alleviating ALI (Li et al., 2014), which is consistent with our findings. Our findings imply that HDN supplementation successfully reduced the harm that D-GalN/LPS did to liver cells.

It is well known that D-GalN/LPS-induced ALI is mainly related to oxidative stress and oxidation-induced secondary inflammation, while hesperidin has been shown to have good antioxidant properties (Zhang et al., 2022; Huang et al., 2024). Shi et al. (2022) demonstrated that HDN can reduce the liver injury induced by LPS in mice. This protective effect is related to the inhibition of oxidative stress and the release of inflammatory mediators, as well as the inhibition of HMGB1 expression and release and the activation of T cells. MDA is a major active aldehyde, mainly derived from the peroxidation of polyunsaturated fatty acids triggered by reactive oxygen species, so it is widely used to reflect the severity of liver injury (Imperatrice et al., 2022). SOD and CAT are important antioxidant enzymes in the body, which can remove superoxide anion free radicals in the body and protect cells from oxidative stress damage. In the liver, SOD and CAT activity is essential for maintaining the normal function and structure of liver cells (Ruart et al., 2019; Guo H. et al., 2019). Our study found that HDN treatment can increase the activity of antioxidant enzymes SOD and CAT, and down-regulate the elevated level of MDA in serum. Previous studies have shown that D-GalN/LPS can activate liver macrophages and induce the production of multiple inflammatory cytokines, which play a key role in the development of ALI, among which IL-1β, IL-6 and TNF-α are classic markers of inflammatory response (Yang et al., 2022). In our study, the contents of inflammatory factors IL-1β, IL-6 and TNF-α were significantly increased after D-GalN/LPS induced acute liver injury in mice, while the contents of these inflammatory factors were significantly decreased after HDN, suggesting that HDN can alleviate the inflammatory response of mouse liver tissue. Above results indicate that HDN has potential antioxidant and anti-inflammatory effects in D-GalN/LPS-induced ALI, which is consistent with previous studies.

In D-GalN/LPS induced acute liver injury model, intestinal barrier dysfunction is often associated with liver injury (Ren et al., 2021). This disorder may be caused by a variety of factors, including the release of inflammatory mediators and the activation of the intestinal immune system. These factors work together to damage the integrity of the intestinal barrier and increase intestinal permeability (Li et al., 2024). Guo K. et al. (2019) have shown that hesperidin can improve tight junction protein expression and intestinal permeability, as well as increase Treg population and restore intestinal barrier function, thus fighting intestinal inflammation. To confirm the dysfunction of intestinal barrier function in ALI mice, we measured intestinal villus height and tight junction protein expression levels. Our study found that D-GalN/LPS treatment resulted in a decrease in the villus height, pillow-to-occludin ratio and ileum microvilli height, and a decrease in the gene expression of ileum tight junction proteins *ZO-1*, *Claudin-1* and *Occludin*. HDN has the potential to restore intestinal mucosal tight junctions and barrier integrity in ALI.

Impaired intestinal barrier function and increased permeability may also lead to bacterial translocation, which in turn causes intestinal flora imbalance (Li et al., 2022). Many studies have shown that ALI is closely related to the imbalance of gut microbiota. The liver, as the first organ to be exposed to microbial products entering the portal circulation, may be affected multiple times by the gut microbiota and its changes (Gong et al., 2018). Recent studies have shown that gut microbiota plays an important role in D-GALN/LPSS-induced acute liver injury in mice. Gut microbiota can inhibit LPS displacement and liver inflammation by regulating and interacting with the gut-liver axis, thereby reducing liver injury (Guan et al., 2021). In this study, 16S rRNA sequencing was used to analyze the gut microbiota of mice in each group. Our study showed significant microbiota disruption, suggesting that ALI can alter the abundance and diversity of gut microbiota, which may directly affect intestinal function. The relative abundance of Lactobacillaceae and bifidobacteriaceae was significantly reduced, which was also seen in acute liver injury models (Adawi et al., 2001; Adawi et al., 1999). Mao et al. (2023) found that the increase of Proteobacteria in CTS-induced liver injury was associated with the disruption of lipid metabolism and the promotion of oxidative stress and liver fibrosis. An increase in Proteobacteria may lead to a breakdown of intestinal barrier function and an increase in intestinal permeability, which allows the release of bacterial endotoxins into the bloodstream, resulting in increased blood endotoxin levels and accumulation in the liver that exceeds the liver’s ability to clear, resulting in liver damage. We found that HDN treatment can increase the richness of Bacteroidetes, reduce the richness of Proteobacteria, restore the integrity of intestinal barrier function, inhibit liver oxidative stress, and thus achieve the prevention and protection of ALI. In previous studies, a mixture of lactobacillus regulates the gut microbiota, increases the amount of short-chain fatty acids (SCFAs), inhibits lipid accumulation and oxidative stress in the liver, improves intestinal epithelial permeability, and reduces LPS entering the portal vein, thereby inhibiting liver inflammation (Estruel-Amades et al., 2019). In order to determine whether HDN induced microbial changes will regulate the metabolic function of the gut microbiota, we conducted functional metagenomic prediction of the gut microbiota based on 16S rRNA gene sequencing. We speculated that the relative abundance of *Lactobacilli* increased after HDN treatment, and a variety of bacteria under *Lactobacilli* belong to probiotics, which are the dominant bacteria in gut microbiota that produce SCFAs, and can maintain the homeostasis of intestinal microflora. Therefore, our results suggest that HDN can alleviate ALI by regulating the composition and structure of the gut microbiota and the production of SCFAs, a metabolite of the intestinal flora, to maintain the integrity of the intestinal barrier, reduce LPS entering the portal vein, and inhibit liver inflammation.

Many evidence indicated that SCFAs are thought to be the products of bacterial fermentation of polysaccharides that benefit the host (Cui et al., 2023). Acetate and butyrate in SCFAs can promote host health by regulating liver function (Shang et al., 2023). HDN intake reversed the decrease in the fecal SCFAs concentration in ALI mice. ALI significantly reduced the abundance of Lachnospiraceae_NK4A136_group, *Alloprevotella*, and *Clostridia_UCG-014*. Lachnospiraceae_NK4A136_group, *Alloprevotella*, and *Clostridia_UCG-014* are also classified as a beneficial bacterium that promotes SCFAs production. This is consistent with a significant decrease of the SCFAs content in the fecal samples of ALI mice. Further, HDN reduced the transfer of LPS to the liver through the portal vein, thereby alleviating hepatic inflammation in mice. HDN upregulates SCFAS to promote body health by regulating intestinal flora. Therefore, HDN alleviates ALI through the gut−liver axis.

Nrf2 is a major regulator of cellular antioxidant responses and can slow the progression of various liver diseases, including viral hepatitis, liver fibrosis, drug-induced liver injury, alcoholic liver disease, and non-alcoholic fatty liver disease (Wang A. et al., 2024; Xun et al., 2021). Upon oxidative stimulation, phosphorylated Nrf2 activates key downstream targets, including HO-1 and NQO1, after being released from Keap1 and transferred to the nucleus by binding to antioxidant response elements. HO-1 and NQO1 are one of the cell protection genes that play a key role in mediating antioxidant, anti-inflammatory and anti-apoptotic effects. In our study, WB and qPCR results showed that D-GalN/LPS-induced acute liver injury in mice inhibited the protein expression of Keap1, Nrf2 and their target genes (SOD, SOD, CAT, GSH-Px, HO-1 and NQO1). All of these inhibition effects can activate the Nrf2/Keap1 signaling pathway by supplementing HDN, and Nrf2 activation increases the expression of antioxidant-related genes, which in turn increases antioxidant capacity. While our study suggests a significant correlation between hesperidin treatment, gut microbiota modulation, and the Nrf2/Keap1 pathway in alleviating liver injury, causality has not been conclusively established. Future studies using gene-editing models and microbiome interventions are warranted to establish a definitive mechanistic link. Overall, our study suggests that dietary supplementation with HDN can alleviate LPS/D-GalNS-induced acute liver injury in mice by modulating the intestinal microbiota to activate the Nrf2/Keap1 signaling pathway.

## 5 Conclusion

This study demonstrates that hesperidin effectively protects against D-GalN/LPS–induced acute liver injury by attenuating inflammation, oxidative stress, and hepatocellular damage. Hesperidin not only restored intestinal barrier integrity but also ameliorated gut microbiota dysbiosis, enriching beneficial microbial taxa associated with metabolic and redox homeostasis (increase the abundance of Lachnospiraceae_NK4A136_group, *Alloprevotella* and *Clostridia_UCG-014*). Mechanistically, activation of the Nrf2/Keap1 pathway played a central role in enhancing antioxidant defenses and reducing hepatic oxidative burden. These findings highlight hesperidin as a promising natural compound capable of modulating the gut–liver axis and strengthening endogenous antioxidant mechanisms, providing valuable insight into its potential therapeutic application in acute liver injury.

## Data Availability

The gut microbiota data presented in this study are deposited in the NCBI repository, accession number PRJNA1366528.
